# Disentangling the effect of illness perceptions on health status in people with type 2 diabetes after an acute coronary event

**DOI:** 10.1186/s12875-018-0720-y

**Published:** 2018-03-02

**Authors:** Rimke Cathelijne Vos, Marise Jeannine Kasteleyn, Monique Johanna Heijmans, Elke de Leeuw, François Georges Schellevis, Mieke Rijken, Guy Emile Rutten, on behalf of the Diacourse study group, Kees J. Gorter, Anne L. van Puffelen, Lianne de Vries, Amber A. W. A. van der Heijden, Caroline A. Baan, Giel Nijpels

**Affiliations:** 1 Julius Center for Health Sciences and Primary Care, University Medical Center Utrecht, Utrecht University, Utrecht, the Netherlands; 2 NIVEL (Netherlands institute for health services research), Utrecht, the Netherlands; 3 Department of general practice and elderly care medicine/EMGO Institute for health and care research, VU University Medical Center, Amsterdam, the Netherlands

**Keywords:** Type 2 diabetes, Multimorbidity, Illness perceptions, Health status, Acute coronary event

## Abstract

**Background:**

Chronically ill patients such as people with type 2 diabetes develop perceptions of their illness, which will influence their coping behaviour. Perceptions are formed once a health threat has been recognised. Many people with type 2 diabetes suffer from multimorbidity, for example the combination with cardiovascular disease. Perceptions of one illness may influence perceptions of the other condition. The aim of the current study was to evaluate the effect of an intervention in type 2 diabetes patients with a first acute coronary event on change in illness perceptions and whether this mediates the intervention effect on health status. The current study is a secondary data analysis of a RCT.

**Methods:**

Two hundred one participants were randomised (1:1 ratio) to the intervention (*n* = 101, three home visits) or control group (*n* = 100). Outcome variables were diabetes and acute coronary event perceptions, assessed with the two separate Brief Illness Perceptions Questionnaires (BIPQs); and health status (Euroqol Visual Analog Scale (EQ-VAS)). The intervention effect was analysed using ANCOVA. Linear regression analyses were used to assess whether illness perceptions mediated the intervention effect on health status.

**Results:**

A positive intervention effect was found on the BIPQ diabetes items *coherence* and *treatment control* (F = 8.19, *p* = 0.005; F = 14.01, *p* < 0.001). No intervention effect was found on the other BIPQ diabetes items *consequence, personal control, identity, illness concern* and *emotional representation*. Regarding the acute coronary event, a positive intervention effect on *treatment control* was found (F = 7.81, *p* = 0.006). No intervention effect was found on the other items of the acute coronary event BIPQ. Better diabetes coherence was associated with improved health status, whereas perceiving more treatment control was not. The mediating effect of the diabetes perception ‘coherence’ on health status was not significant.

**Conclusion:**

Targeting illness perceptions of people with diabetes after an acute coronary event has no effect on most domains, but can improve the perceived understanding of their diabetes. Discussing perceptions prevents people with type 2 diabetes who recently experienced an acute coronary event from the perception that they will lose control of both their diabetes and the acute coronary event. Illness perceptions of diabetes patients should therefore be discussed in the dynamic period after an acute coronary event.

**Trial registration:**

Nederlands trial register; NTR3076, Registered September 20 2011.

## Background

Patients with a chronic disease such as type 2 diabetes develop perceptions of their illness. It is assumed that perceptions are formed once a health threat has been recognised [1]. Illness perceptions include thoughts regarding the *cause(s)* of the illness, perceived symptoms attributed to the illness (*identity*), the *timeline*, the perceived *consequences*, the perceived amount of *control* patients feel over the illness and thoughts regarding the helpfulness of treatment (*treatment control*) [1]. Later research added the perceived understanding of the illness (*coherence)* and *emotional representation* to the model [2]. These dimensions make up the individual’s overall illness perception and may have an important impact on coping, feelings of self-efficacy and psychological well-being [3, 4].

Short-term interventions can alter illness perceptions and correct misconceptions, and, as a result, improve health related behaviours and outcomes [4–6]. As illness perceptions may change due to new developments or information, discussing them might be important in a period of influential changes in an individual’s health situation [1, 7]. When persons with type 2 diabetes are confronted with comorbidities they will develop perceptions of the comorbidities and former perceptions of diabetes might change [8].

Type 2 diabetes patients are mostly unaware of their increased risk of cardiovascular comorbidity and often do not attribute the occurrence of an acute coronary event to their diabetes [8–10]. If they experience such an event, they perceive themselves as different from other coronary patients, since they have to cope with two conditions at the same time [11, 12].

Diabetes-related distress may increase in patients with type 2 diabetes after a first acute coronary event [13–15]. Because positive illness perceptions are associated with decreased distress [4, 16], we hypothesised that discussing illness perceptions after an acute coronary event could be beneficial. Evidence on the effectiveness of interventions for people with the combination of an acute coronary event and type 2 diabetes is scarce [17].

We developed an intervention, consisting of three home visits by a diabetes nurse, for type 2 diabetes patients, shortly after their first acute coronary event [18]. During these visits, illness perceptions of both diabetes and the acute coronary event were discussed. We hypothesised that improving illness perceptions would lead to better self-management which would decrease diabetes-related distress. The main results of this randomised controlled trial (RCT) have been published previously and show that although the intervention had no effect on diabetes-related distress, which was the primary outcome, it improved health status and had a positive effect on diabetes illness perceptions [19].

To explore the role of illness perceptions in more detail we performed a secondary analysis aiming to describe the effect of the intervention on the different domains of illness perceptions. We examined the effects on patients’ perceptions of their type 2 diabetes and the acute coronary event, and evaluated whether a change in perception(s) mediated the intervention effect on health status.

## Methods

### Participants and setting

This study is a secondary analysis of an RCT [19]. Between October 2011 and August 2013 type 2 diabetes patients with a first acute coronary event were recruited via their cardiologists in thirteen hospitals in three regions across the Netherlands. An acute coronary event was defined as a percutaneous transluminal coronary angioplasty (PTCA), a coronary artery bypass graft (CABG) procedure and/or a myocardial infarction (MI). After hospital discharge, individuals were invited to participate if 1) they were known with type 2 diabetes for more than one year; 2) were older than 35 years; 3) had sufficient knowledge of the Dutch language; 4) were able to fill in questionnaires; and 5) had no other serious illnesses or conditions that would prevent full participation. Exclusion criteria were 1) having had a previous acute coronary event and 2) being discharged from the hospital longer than two weeks. After informed consent, participants were randomised in a 1:1 ratio (parallel groups) to the intervention or the attention control group [20]. Randomisation, stratified on region, was generated at the person level by a computerized random-number generator at the research centre. The study protocol was registered at the trial registration (Nederlands trial register; NTR3076) and approved by the Medical Ethical Committee of the University Medical Center Utrecht (Protocol number 10–403).

### Intervention and control group

The full details of the design, rationale and theoretical framework of this RCT have been described previously [18]. The intervention aimed to strengthen beneficial illness perceptions and alter less beneficial perceptions, with the purpose to decrease diabetes-related distress and improve health status. For example, both type 2 diabetes and the acute coronary event were discussed during the intervention and questions about the (consequences of) both diseases were considered in order to increase coherence. Furthermore, feelings of uncontrollability of the conditions were discussed to identify aspects of the disease that could be controlled. Seven trained diabetes nurses visited the participants in the intervention group three times at their homes (Table 1). During the visits, the nurses used a questionnaire on illness perceptions and strategies of motivational interviewing to stimulate beneficial perceptions of both type 2 diabetes and the coronary event, and they tried to challenge less beneficial conceptions.

**Table 1:** Characteristics of the tailored supportive intervention

Visit nr	Components	Description
Visit: 1 Moment: < 3 weeks after discharge Aimed duration: 65 min	Problem mapping	The patient indicated to what extent problems were experienced from a list of ten topics: 1. physical activity; 2. Sexuality; 3. Pharmacotherapy; 4. monitoring scheme with different health care professionals; 5. coping together with the partner; 6. coping with the diabetes in daily life; 7. coping with an acute coronary event in daily life; 8. (depressive) feelings; 9. nutrition/diet; 10. other problems.
	Discussion in depth	The nurse, patient and partner discussed in depth the three topics that were considered by the patients most important;
	Goal setting	The patient set goals he/she wanted to achieve in the following two weeks.
	Home work	The patient was asked to keep a daily log to track strategies for coping with events related to the topics discussed. To adjust possible less beneficial perceptions regarding diabetes, the acute coronary event and their relationship. Patients’ were asked to complete the BIPQ again. These answers were only used by the nurse as guide to discuss illness perceptions during the second visit and not as outcome.
Visit: 2 Moment: 2 weeks after first visit Aimed duration: 45 min	Discussion in depth	Problems discussed during the first visits were reviewed to explore the remaining difficulties. The daily log was discussed to explore the strategies the patient used to cope with problems.
	Discussion of illness perceptions	The reported illness perceptions were discussed to challenge misconceptions of diabetes and the acute coronary event.
	Goal setting	The goals formulated during the first visit were evaluated and new goals were formulated.
	Home work	The patient was asked to use a weekly log to track strategies for coping with difficulties.
Visit: 3 Moment: 2 months after second visits Aimed duration: 45 min	Discussion in depth	Problems and illness perceptions which were discussed during the second visit were reviewed to explore the remaining difficulties on these topics. The weekly log was discussed to explore the strategies the patients used to cope with problems.
	Problem mapping	The patient indicated again to what extent problems were encountered on the list of topics from the first visit and strategies to cope with these topics were discussed.
	Goal setting	The goals formulated during the second visit were evaluated, and new goals were formulated.
	Discussion about future	At the end of the visit uncertainties for the future regarding coping with the acute coronary event and type 2 diabetes were discussed.

Participants in the attention control group received one consultation (approximately 15 min) by telephone, within three weeks after hospital discharge, to discuss how they felt and functioned in the period after discharge. Two members of the research team conducted the consultations, in accordance with a semi structured protocol. They were instructed to give the patients personal attention, without giving advice or support regarding treatment and self-management. The focus of the consultation was on the topics which were of high importance to the patient. Open questions were used to stimulate the discussion (e.g. “Can you tell me how you have been doing since you got home?”).

### Outcome variables

Patients completed a set of questionnaires at their home, after hospital discharge but before the first home visit/phone consultation (T0) and five months later (T1). The validated Dutch version of the Brief Illness Perception Questionnaire (BIPQ) was used to measure illness perceptions [21]. It comprises eight items on a 0–10 scale on the dimensions: 1. *Consequences* (“How much does your illness affect your life?”); 2. *Timeline* (“How long do you think your illness will continue?”); 3. *Personal control* (“How much control do you feel you have over your illness?”); 4. *Treatment control* (“How much do you think your treatment can help your illness?”); 5*. Identity* (“How much do you experience symptoms from your illness?”); 6. *Illness concern* (“How concerned are you about your illness”); 7. *Coherence* (“How well do you feel you understand your illness?”); and 8. *Emotional representation* (“How much does your illness affect you emotionally?”). Regarding the beneficial and less beneficial perceptions, it is known that people who perceive little control over and coherence of their disease have a lower quality of life [16]. In addition, an open question assessed the perceived cause(s) of illness, by which respondents list their three most important factors they believe to have caused their illness. The BIPQ shows good test-retest reliability and is designed as a one-item domain instrument [22]. At baseline and follow-up participants received a version of the BIPQ that measured their perceptions of the diabetes (diabetes-BIPQ) and a version that measured their perceptions of the acute coronary event (acute coronary event-BIPQ).

Self-reported health status was measured using the Euroqol Visual Analogue Scale (EQ-VAS) [23], a subjective measurement of health status, measured on a graded vertical line ranging from 0 to 100. Participants were asked to mark a point on the EQ-VAS that best reflected their actual health status; a score of 100 representing the best and a score of 0 the worst imaginable health state.

Data about HbA1c levels, other comorbidities (cancer, chronic respiratory diseases, joint problems, other) and microvascular complications (eye problems, kidney problems, neurological problems and foot problems) were extracted from hospital files.

### Statistical analysis

For the analyses described in this manuscript, we based our post-hoc calculation on health status measured with the EQ-VAS. With a sample size of 161, a mean score of 69.5 on the EQ-VAS, a standard deviation of 16.4, a two-sided significance level of 5% and a power of 80% we were able to detect a difference of 7.0 on the EQ-VAS. The minimal important difference of the EQ-VAS is assumed to be > 3.0 [24]. Therefore, our sample size of 161 was large enough to detect a clinically relevant difference on the EQ-VAS.

Data were analysed according to the intention-to-treat principle. Thus, participants who did not complete the intervention, or did not want the telephone consultation, were asked to complete the follow-up questionnaires as well. Descriptive statistics were used to describe the study sample. Changes in illness perceptions were analysed using ANCOVA, for the items of the diabetes-BIPQ and acute coronary event-BIPQ separately. In the model, treatment allocation (intervention or control group) was included as factor and the baseline score on the BIPQ item as covariate.

For the illness perceptions items that were influenced by the intervention, we assessed their potential mediating role to explain the intervention effect on participants’ health status by estimating a series of linear regression models as proposed by Baron and Kenny [24]. In a first model the effect of the independent variable (here, the intervention) on the mediator (here, change in an illness perception variable from baseline to 5 months later) was estimated. In a second model the effect of the independent variable (here, the intervention) on the dependent variable (here, change in health status from baseline to 5 months later) was estimated. And in the final, third model, the effects of both the independent variable (intervention) and the mediator (change in an illness perception variable) on the dependent variable (change in health status) were estimated.

**Fig. 1:**
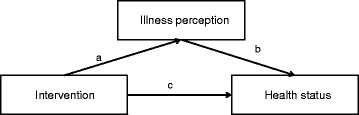
The mediating role of illness perceptions in the effect of the intervention on health status. Figure adjusted from Baron and Kenny [24]

Following Baron and Kenny, mediation by participants’ illness perception is established, if the intervention has a significant effect on participants’ illness perception(s) in the first model and on their health status in the second model, and participants’ illness perception(s) significantly affects their health status in the third model. This mediation is illustrated in Fig. 1, by the causal pathway Intervention → Illness perception (path a) → Health status (path b). The figure shows that the mediation effect is an indirect effect of the intervention on health status via its impact on participants’ illness perception(s). If the indirect effect is significantly different from zero (using Sobel’s test, as recommended by Baron and Kenny [24]) we conclude that the intervention effect on health status is mediated by participants’ illness perception(s). A *p*-value of *p* < 0.05 was considered significant. All analyses were performed using SPSS version 20.0.

All analyses were performed using SPSS version 20.0.

## Results

**Fig. 2:**
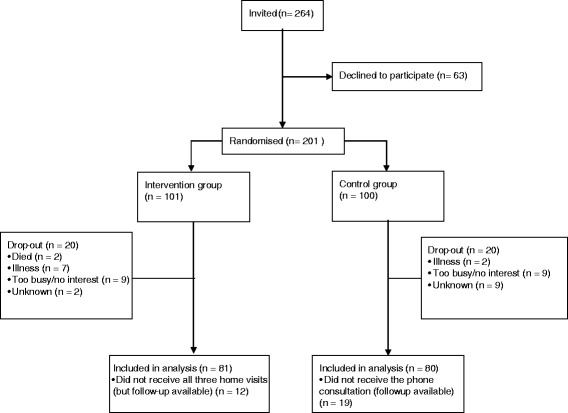
Flow-diagram of patient enrolment, allocation and analysis

A total of 264 individuals were invited to participate, of which 24% (*n* = 63) declined (Fig. 2). As a result, 201 individuals were randomly allocated to either the intervention or the attention control group.

Follow-up data were available of 81 intervention and 80 control group participants and were included in the analysis; their characteristics are presented in Table 2. Dropouts were more often female (50 vs. 25%; *p* = 0.001), more likely to be living alone (35 vs. 18%; *p* = 0.015) or to have microvascular complications (41 vs. 24%; *p* = 0.045). No significant differences between dropouts and completers were found with regard to baseline scores on items of the BIPQ and EQ-VAS. Lifestyle factors (76%) were most often indicated as one of the three most important causes of the acute coronary event, followed by genetics (27%) and having an increased cardiovascular risk because of being diagnosed with hypertension, lipid disorder or atherosclerosis (24%) (Table 2). About a quarter (22%) of the patients indicated their type 2 diabetes as one of the most important causes. In both the intervention and control group participants were well controlled, and no significant differences in HbA1c were found between baseline and follow-up within or between groups (F = 0.11, *p* = 0.745).

**Table 2:** Baseline characteristics of intervention and control group

	Intervention group (*n* = 81)	Control group (*n* = 80)	Total (*n* = 161)
Male gender, *n* (%)	62 (76.5)	60 (75.0)	122 (75.8)
Age, years (SD)	66.0 (9.3)	65.6 (9.4)	65.8 (9.3)
Living alone, *n* (%)	14 (17.3)	16 (20.0)	30 (18.6)
Duration of type 2 diabetes, years (IQR)	7.0 (2.8–16.0)	8.5 (5–15)	8.0 (4–15)
Use of oral diabetes medication, *n* (%)	67 (82.7)	66 (82.5)	133 (82.6)
Use of insulin, *n* (%)	28 (34.6)	23 (28.8)	51 (31.7)
HbA1c, mmol/mol (SD)	55.0 (15.4)	51.0 (10.6)	53.6 (13.4)
Smoking status			
Never, *n* (%)	19 (23.5)	20 (25.0)	39 (24.2)
Former, *n* (%)	55 (67.9)	53 (66.3)	108 (67.1)
Current, *n* (%)	7 (8.6)	7 (8.8)	14 (8.7)
Type of acute coronary event			
CABG with/without MI, *n* (%)	14 (17.3)	16 (20.0)	30 (18.6)
PTCA with/without MI, *n* (%)	53 (65.4)	47 (58.8)	100 (62.1)
MI or instable angina pectoris without invasive intervention, *n* (%)	14 (17.3)	17 (21.3)	31 (19.2)
Comorbidity			
Cancer, *n* (%)	9 (11.1)	6 (7.5)	15 (9.3)
Respiratory illness, *n* (%)	9 (11.1)	9 (11.3)	18 (11.2)
Joint problems, *n* (%)	9 (11.1)	12 (15.0)	21 (13.0)
Other, *n* (%)	13 (16.0)	11 (13.8)	24 (14.9)
Microvascular complications			
Eye problems, *n* (%)	13 (16.0)	6 (7.5)	19 (11.8)
Kidney problems, *n* (%)	8 (9.9)	6 (7.5)	14 (8.7)
Neurologic problems, *n* (%)	4 (4.9)	1 (1.3)	5 (3.1)
Diabetic foot abnormalities, *n* (%)	5 (6.2)	1 (1.3)	6 (3.7)
Followed cardiac rehabilitation, *n* (%)	41 (50.1)	46 (57.5)	87 (54.0)
Causal item type 2 diabetes-IPQ			
Increased cardiovascular risk, n (%)	2 (2.5)	3 (3.8)	5 (3.1)
Genetics, n (%)	25 (30.9)	24 (30.0)	49 (30.4)
Lifestyle, n (%)	47 (58.0)	43 (53.8)	90 (55.9)
Stress, n (%)	11 (13.6)	9 (11.3)	20 (12.4)
Age, n (%)	6 (7.4)	15 (18.8)	21 (13.0)
Medication use, n (%)	3 (3.7)	1 (1.3)	4 (2.5)
Don’t know, n (%)	8 (9.9)	9 (11.3)	17 (10.6)
Other, n (%)	10 (12.3)	13 (16.3)	23 (14.3)
Causal item acute coronary event-IPQ			
Type 2 diabetes, *n* (%)	18 (22.3)	18 (22.5)	36 (22.4)
Increased cardiovascular risk, *n* (%)	23 (28.4)	15 (18.8)	38 (23.6)
Genetics, *n* (%)	24 (29.6)	20 (25.0)	44 (27.3)
Lifestyle, *n* (%)	56 (69.1)	67 (83.8)	123 (76.4)
Stress, *n* (%)	14 (17.3)	22 (27.5)	36 (22.4)
Age, *n* (%)	1 (1.2)	4 (5.0)	5 (3.1)
Incident, *n* (%)	2 (2.5)	1 (1.3)	3 (1.9)
Don’t know, *n* (%)	10 (12.3)	5 (6.3)	15 (9.3)
Other, *n* (%)	11 (13.6)	16 (0.2)	27 (16.8)

### Effects of the intervention on the perceptions of diabetes

**Fig. 3:**
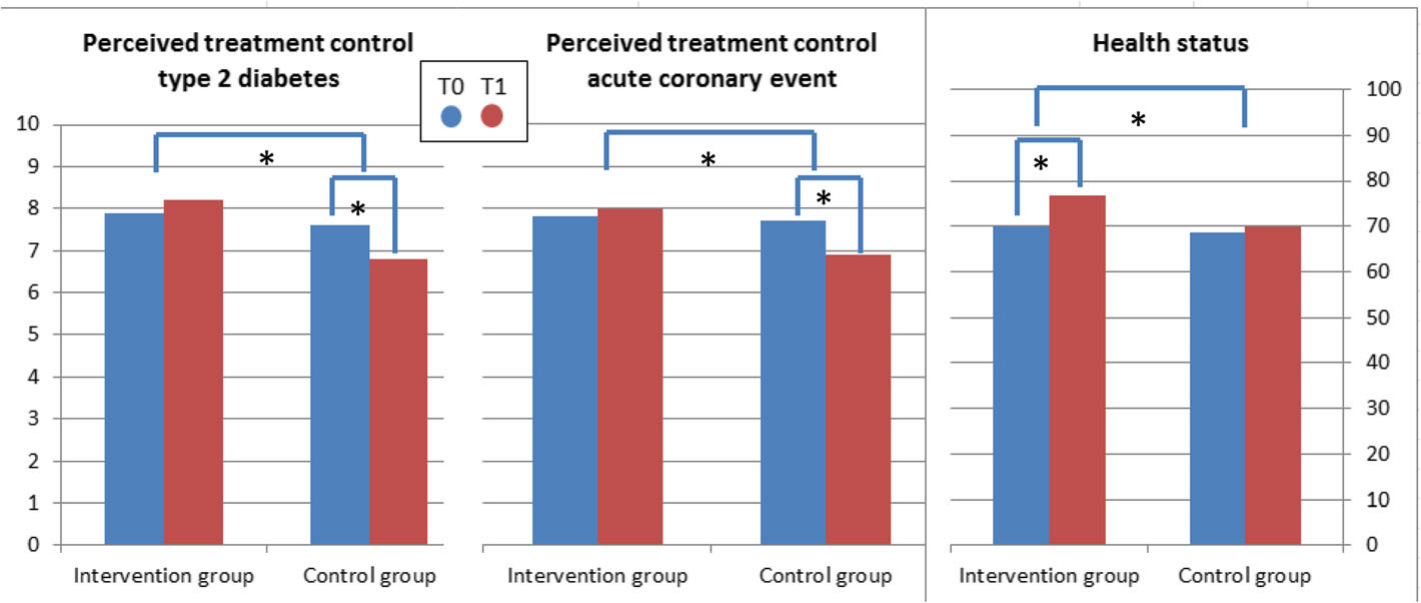
Intervention effect on the perceived treatment control of type 2 diabetes, the perceived treatment control of the acute coronary event and health status * *p* < 0.05

Mean scores on the diabetes-related perceptions at baseline and follow-up are presented in Table 3. A significant intervention effect on *coherence* (F = 8.19, *p* = 0.005) was found; only participants in the intervention group perceived a better understanding of their diabetes at follow-up compared to baseline (T0: 6.5 ± 2.9; T1: 7.3 ± 2.5, *p* = 0.003). A significant intervention was also found on perceived treatment control of the diabetes (F = 14.01, *p* < 0.001); the participants in the control group showed decreased feelings of treatment control after five months (T0: 7.6 ± 2.0; T1: 6.7 ± 2.3, *p* = 0.007), while these perceptions did not change in the intervention group (Fig. 3).

**Table 3:** Baseline and follow-up scores on the type 2 diabetes BIPQ (Mean scores (SD))

	Intervention group *N* = 81		Control group *N* = 80		Intervention effect	
	T0	T1	T0	T1	F	P
Consequence	3.7 (3.0)	3.5 (2.8)	4.0 (3.0)	3.9 (2.9)	0.28	0.596
Timeline	9.4 (1.8)	9.3 (1.8)	9.2 (2.1)	9.2 (2.1)	0.00	0.982
Personal control	6.5 (2.5)	6.8 (2.6)	7.2 (2.1)	6.8 (2.3)	1.06	0.304
Treatment control	7.8 (2.0)	8.0 (1.9)	7.6 (2.0)	6.7 (2.3)*	14.01	**< 0.001**
Identity	3.4 (3.1)	3.3 (3.0)	3.7 (3.1)	3.6 (2.8)	0.05	0.822
Illness concern	3.8 (2.9)	3.7 (2.9)	4.2 (3.3)	4.0 (3.0)	0.02	0.879
Coherence	6.5 (2.9)	7.3 (2.5)*	6.4 (2.9)	6.3 (2.8)	8.19	**0.005**
Emotional representation	2.8 (2.7)	3.1 (2.8)	3.0 (3.1)	3.0 (2.9)	0.26	0.610

*The boldface entries correspond to a significant effect within a group

### Effects of the intervention on the perceptions of an acute coronary event

Regarding the perceptions of an acute coronary event, an intervention effect was found on perceived treatment control of (F = 7.81, *p* = 0.006) (Table 4). Again, perceptions of treatment control decreased in the control group (7.5 ± 2.0; T1: 6.9 ± 2.6, *p* = 0.036), but not in the intervention group. Furthermore, the perceived personal control over the coronary morbidity significantly improved in the intervention group (T0: 4.8 ± 2.7; T1: 5.6 ± 2.9, *p* = 0.004) and did not change in the control group, but the intervention effect was not significant. Furthermore, the control group had less concerns regarding the acute coronary event at follow-up compared to directly after discharge (T0: 5.6 ± 3.0; T1: 4.9 ± 2.8, *p* = 0.020), while concerns did not change in the intervention group (Fig. 3).

**Table 4:** Baseline and follow-up scores on the type acute coronary event BIPQ (Mean scores (SD))

	Intervention group *N* = 81		Control group *N* = 80		Intervention effect	
	T0	T1	T0	T1	F	P
Consequence	5.8 (3.3)	5.3 (3.1)	6.2 (2.9)	5.8 (2.9)	0.31	0.581
Timeline	6.5 (3.5)	7.0 (3.6)	6.7 (3.5)	7.3 (3.3)	0.08	0.776
Personal control	4.8 (2.7)	5.6 (2.9)*	5.3 (2.7)	5.6 (2.9)	0.89	0.347
Treatment control	7.8 (2.2)	8.0 (2.1)	7.5 (2.0)	6.9 (2.6)*	7.81	**0.006**
Identity	3.9 (3.1)	3.5 (2.9)	4.8 (2.8)	4.3 (2.7)	0.77	0.381
Illness concern	5.1 (3.3)	4.9 (3.4)	5.6 (3.0)	4.9 (2.8)*	0.76	0.385
Coherence	6.3 (3.0)	6.8 (2.9)	6.3 (2.7)	6.6 (2.8)	0.46	0.499
Emotional representation	4.1 (3.1)	3.8 (3.0)	4.3 (3.1)	3.9 (3.0)	0.05	0.830

*The boldface entries correspond to a significant effect within a group

### Mediating effect of illness perceptions on the intervention effect on health status

**Fig. 4:**
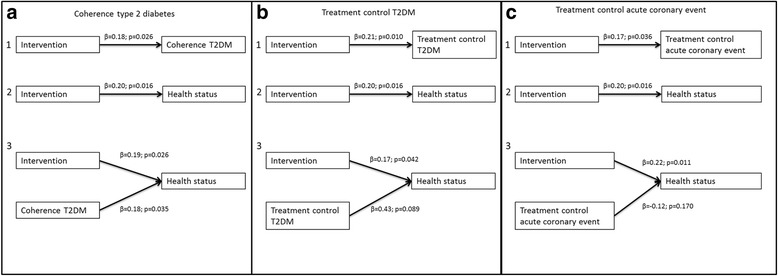
The mediating effect of illness perceptions on health status

A significant improvement on health status was found after the intervention (T0: 69.9 ± 17.3; T1:76.8 ± 15.6, *p* < 0.001) as well as a significant intervention effect (β = 0.20, *p* = 0.016; Fig. 4a–c).

Regarding the effects of participants’ change in illness perceptions on their health status, few significant effects were found. A better perceived coherence regarding the diabetes was associated with a better health status (β = 0.18, *p* = 0.035). The effect of the intervention on health status was slightly lower when coherence regarding the diabetes was taken into account compared to the model without coherence (Fig. 4a). Nevertheless, the mediating effect was not significant, as demonstrated by the Sobel z-test (z’ = 1.54, *p* = 0.12). The total-, direct- and indirect effects are reported in Table 5. Perceived treatment control regarding the diabetes (Fig. 4b) and perceived treatment control regarding the acute coronary event (Fig. 4c) were not associated with health status and hence were not considered to act as mediators.

**Table 5:** Mediating effect of coherence of type 2 diabetes on the intervention effect on health status

	β	*p*-value
Effect of the intervention on coherence^a^	0.18	0.026
Total effect of the intervention on health status^b^	0.20	0.016
Effect of change in coherence on health status^c^	0.18	0.035
Direct effect of the intervention on health status^c^	0.19	0.026
Indirect effect of the intervention on health status^d^	0.01	

β is the standardized regression coefficient

^a^ model 1: The effect of the intervention on diabetes coherence

^b^ model 2: The effect of the intervention on health status

^c^ model 3: the effect of the intervention and change in diabetes coherence on health status

^d^ indirect effect = total effect – direct effect

## Discussion

This study assessed the impact of a tailored supportive intervention for type 2 diabetes patients shortly after hospital discharge after a first acute coronary event on their illness perceptions and the mediating effect of these perceptions on the improved health status. Five months after discharge from hospital people who had not received the intervention had lower perceptions of treatment control regarding their diabetes and their heart disease compared to immediately after discharge. This decline was prevented by the intervention, since the perceived treatment control did not change in the intervention group. In other words, five months after discharge, people that had not received the intervention perceived that treatment would be less helpful than at baseline. Although we expected that this change in treatment control would have an effect on health status, no such association was found. Patients perceived a better understanding of their diabetes after the intervention, but this could not explain the positive effect on their health status.

In type 2 diabetes, perceived treatment control is associated with better medication adherence [25]. Individuals with type 2 diabetes and an acute coronary event experience problems with coping with the amount of medication and their adverse effects [12]. They are at increased risk for medication non-adherence, in particular when their perceived treatment control is low [26]. Medication non-adherence is indeed prevalent among individuals with diabetes and cardiovascular disease and is associated with adverse outcomes [27]. People who see their coronary event as less controllable have an increased risk for a new cardiac complication [28].

After the tailored supportive intervention, participants had a better understanding of their diabetes, but not of their heart disease, which might be explained by the fact that participants in the intervention group received home visits by a diabetes nurse. Although these nurses were trained on the cardiovascular topics, it is likely they could support participants in particular on diabetes understanding, since that is their expertise. Furthermore, all patients will most likely receive a lot of information about the coronary event during their hospital stay.

Health care providers are aware of the increased risk of cardiovascular morbidity in type 2 diabetes patients and report to discuss this with them [29]. However, in our study only 22% of the participants mentioned their type 2 diabetes as cause of the coronary event, indicating that most of them experience the acute coronary event as comorbidity rather than as complication. When multimorbidity is present, people are likely to prioritise other (more recent) conditions above their diabetes [30].

Our study showed that illness perceptions did not mediate the intervention effect on health status, suggesting that other factors play a role. Next to illness perceptions, self-efficacy was a central psychological concept in the intervention [18]. According to the Social Cognitive theory, self-efficacy is modifiable and can affect health status [31] Although the intervention focussed on self-efficacy, no intervention effect on self-efficacy was found, and therefore this concept could also not explain the intervention effect [19]. Nevertheless, it should be noted that only the effect on diabetes self-efficacy was measured and tested. Therefore, it could be thought that general self-efficacy or self-efficacy regarding the acute coronary event act as mediator, since the intervention not only focussed on diabetes self-efficacy.

A potential limitation of this study is that the hospital based nurses invited eligible patients; It is possible that they excluded patients whom they did not find suitable for the study. It could also be that patients hospitalised for a shorter period were missed, because they were discharged before they received an invitation letter. This might have caused selection bias. Nevertheless, participants were comparable to other patients regarding age, gender and treatment (PTCA, CABG or non-invasive intervention) of the acute coronary event [32], which suggests that they are representative in terms of severity of their heart disease for all patients with an acute coronary event in the Netherlands. However, we cannot be sure whether our population is representative in terms of education, health literacy and cultural factors, which might all be associated with illness perceptions. This may limit the generalizability of our results.

Another limitation might be the short follow-up period, which limits the possibility to determine the sustainability of the treatment effect. Furthermore, one should note that participants completed the BIPQ first with regard to their diabetes, followed by the same items for the acute coronary event. Because it can be difficult to ascribe symptoms or medications to a specific disease this could result in overlapping perceptions of multiple illnesses [33]. Using disease-specific questionnaires in people with multiple conditions may result in not capturing all problems encountered by the group of patients.

Furthermore, statisticians have identified problems with the Baron and Kenny approach [34–37]. One of the shortcomings is that a significant total effect should be demonstrated before continuing with the test for mediation, although there are situations where mediation occurs even in the absence of a total effect [37]. Another shortcoming is that it requires that the previously significant total effect becomes nonsignificant [38]. Therefore, it can be thought that in our study there is still a (partly) mediating effect of illness perceptions, although this could not be demonstrated with the Baron and Kenny method.

Furthermore, the current study was not designed to assess the mediating effect of illness perceptions of the intervention on health status. The primary outcome of the study was diabetes related distress [18]. Although no effect was found on distress, a positive effect on health status was found [19]. Since we hypothesised that this improvement in health status could be explained by a change in illness perceptions we evaluated this in the current study, although not planned before designing the RCT. Finally, it could be thought that the change in illness perceptions could be explained by a change in health status. According to the common sense model, perceptions of individuals are formed by their own experiences, including changes in perceived health [1]. For the individuals included in this study, an improvement in perceived health could be expected over time, which in turn might influence illness perceptions. Still, such a change could be expected in both the intervention and control group where we only found an improvement in the intervention group.

## Conclusion

An intervention focussing on illness perceptions improves health status of type 2 diabetes patients after their first acute coronary event. Furthermore, discussing illness perceptions in the dynamic period after an acute coronary event can improve their perceived understanding of diabetes. In addition, discussing patients’ illness perceptions helps to maintain the perceived controllability of the diabetes and the heart disease. Nevertheless, these findings on illness perceptions did not explain the intervention effect on health status. Other illness perceptions did not change over time in the period after discharge from hospital after a first acute coronary event or as a result of the intervention.

## Abbreviations

BIPQ: Brief Illness Perceptions Questionnaire
CABG: Coronary Artery Bypass Graft
EQ-VAS: Euroqol Visual Analog Scale
MI: Myocardial Infarction
PTCA: Percutaneous Transluminal Coronary Angioplasty
T2DM: Type 2 Diabetes Mellitus
